# Woolwich Death-Rate Lowest

**Published:** 1919-02-15

**Authors:** 


### Woolwich Death-rate Lowest.

The annual report of Dr. Sidney Davies, medical officer of health for Woolwich, gives some interesting information regarding the health of the borough during 1917. Notwithstanding the adverse conditions arising from the war the health of the borough during last year was remarkably good, as is indicated by the death-rate, which was the lowest of all the Metropolitan boroughs, being 10.6 compared with 12.3 for 1916. As in other districts, the birth-rate shows a marked fall, being 17.1 compared with 21.7 for the previous year. The infant mortality was 79, which is the lowest rate recorded except for 1912 and 1916, and Woolwich occupies the commendable position of having the lowest infantile death-rate of any Metropolitan borough. The zymotic death-rate was low, being 0.69 compared with 0.85 in 1916. There was a slight increase in the death-rate from measles, but a decrease in the death-rate from all other zymotic diseases. No case of smallpox occurred during the year. There were seventeen cases of cerebro-spinal meningitis, the same number as in the previous year, and of this number five terminated fatally. There was a slight increase in the number of deaths from influenza, and while there was a fall in the number of deaths from bronchitis and pneumonia in adults, an increase in the deaths from these causes under five years, probably due to the increased mortality from measles, is recorded. It is satisfactory to note that the death-rate from pulmonary tuberculosis is lower, the rate being 1.17 compared with 1.22 for the previous year. This is a somewhat exceptional circumstance, and is one upon which the health authorities of the borough of Woolwich deserve to be congratulated. An important investigation has been carried out by Dr. Davies with regard to the habits of persons suffering and dying from malignant disease, and the results are given in tabulated form. The habits with reference to which inquiries were made are as follows: excessive alcohol drinking, smoking and tea drinking, excessive eating and meat eating, and constipation. Excessive alcohol drinking and smoking were present in a considerably higher percentage of the cancer cases than in the non-cancerous control cases. In view of the persistent rise in the death-rate from malignant disease, which has been the feature of recent years, the subject is one which calls for continued investigation.

